# (*Z*)-3-(4-Chloro­benz­yl)-1,5-benzothia­zepin-4(5*H*)-one

**DOI:** 10.1107/S1600536812026608

**Published:** 2012-06-16

**Authors:** D. Lakshmanan, S. Murugavel, R. Selvakumar, M. Bakthadoss

**Affiliations:** a Department of Physics, C. Abdul Hakeem College of Engineering & Technology, Melvisharam, Vellore 632 509, India; b Department of Physics, Thanthai Periyar Government Institute of Technology, Vellore 632 002, India; c Department of Organic Chemistry, University of Madras, Maraimalai Campus, Chennai 600 025, India

## Abstract

In the title compound, C_16_H_12_ClNOS, the seven-membered thia­zepine ring adopts a distorted twisted boat conformation. The dihedral angle between the least-squares planes of the 1,5-benzothia­zepine ring system and the benzene ring is 50.2 (1)°. In the crystal, pairs of N—H⋯O hydrogen bonds link centrosymmetrically related mol­ecules into dimers, generating *R*_2_^2^(8) ring motifs. The crystal packing is further stabilized by π–π inter­actions [centroid–centroid distance = 3.763 (2) Å].

## Related literature

For the pharmaceutical properties of thia­zepin derivatives, see: Tomascovic *et al.* (2000 ▶); Rajsner *et al.* (1971 ▶); Metys *et al.* (1965 ▶). For related structures, see: Sridevi *et al.* (2011 ▶); Sabari *et al.* (2011 ▶); Selvakumar *et al.* (2012 ▶). For ring-puckering parameters, see: Cremer & Pople (1975 ▶). For hydrogen-bond motifs, see: Bernstein *et al.* (1995 ▶).
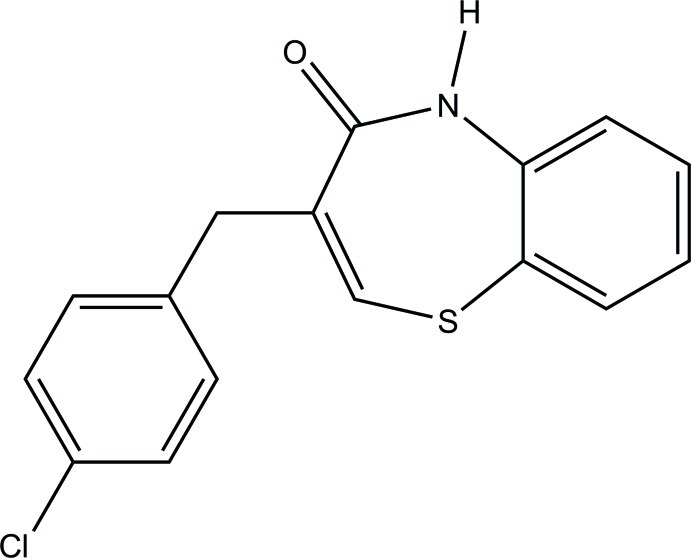


## Experimental

### 

#### Crystal data

C_16_H_12_ClNOS
*M* *_r_* = 301.78
Orthorhombic, 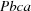

*a* = 9.0486 (3) Å
*b* = 9.4105 (3) Å
*c* = 33.4876 (10) Å
*V* = 2851.53 (16) Å^3^
*Z* = 8
Mo *K*α radiation
μ = 0.41 mm^−1^
*T* = 293 K
0.23 × 0.21 × 0.15 mm

#### Data collection

Bruker APEXII CCD diffractometer
Absorption correction: multi-scan (*SADABS*; Sheldrick, 1996 ▶) *T*_min_ = 0.910, *T*_max_ = 0.941
14850 measured reflections
3099 independent reflections
2382 reflections with *I* > 2σ(*I*)
*R*_int_ = 0.029

#### Refinement

*R*[*F* ^2^ > 2σ(*F* ^2^)] = 0.047
*wR*(*F* ^2^) = 0.118
*S* = 1.03
3099 reflections
182 parameters
H-atom parameters constrained
Δρ_max_ = 0.35 e Å^−3^
Δρ_min_ = −0.45 e Å^−3^

### 

Data collection: *APEX2* (Bruker, 2004 ▶); cell refinement: *APEX2* and *SAINT* (Bruker, 2004 ▶); data reduction: *SAINT* and *XPREP* (Bruker, 2004 ▶); program(s) used to solve structure: *SHELXS97* (Sheldrick, 2008 ▶); program(s) used to refine structure: *SHELXL97* (Sheldrick, 2008 ▶); molecular graphics: *ORTEP-3* (Farrugia, 1997 ▶); software used to prepare material for publication: *SHELXL97* and *PLATON* (Spek, 2009 ▶).

## Supplementary Material

Crystal structure: contains datablock(s) global, I. DOI: 10.1107/S1600536812026608/tk5111sup1.cif

Structure factors: contains datablock(s) I. DOI: 10.1107/S1600536812026608/tk5111Isup2.hkl

Supplementary material file. DOI: 10.1107/S1600536812026608/tk5111Isup3.cml

Additional supplementary materials:  crystallographic information; 3D view; checkCIF report

## Figures and Tables

**Table 1:** Hydrogen-bond geometry (Å, °)

*D*—H⋯*A*	*D*—H	H⋯*A*	*D*⋯*A*	*D*—H⋯*A*
N1—H1⋯O1^i^	0.86	2.07	2.854 (2)	151

Symmetry code: (i) 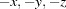.

**Fig. 1.**
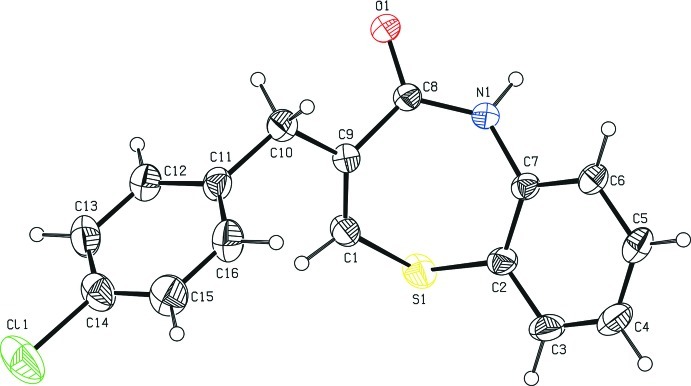
Molecular structure of the title compound showing displacement ellipsoids at the 30% probability level. H atoms are presented as a small spheres of arbitrary radii.

**Fig. 2.**
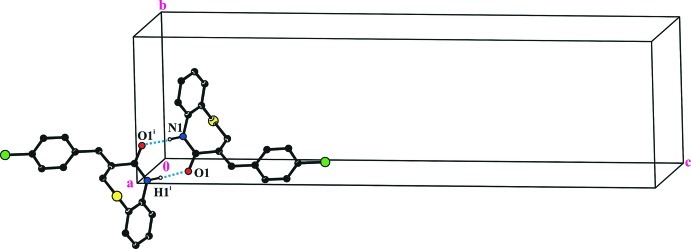
Part of the crystal structure of the title compound showing N—H···O intermolecular hydrogen bonds (dotted lines) generating an R22(8) centrosymmetric dimer [Symmetry code: (i) -x, -y, -z].

**Fig. 3.**
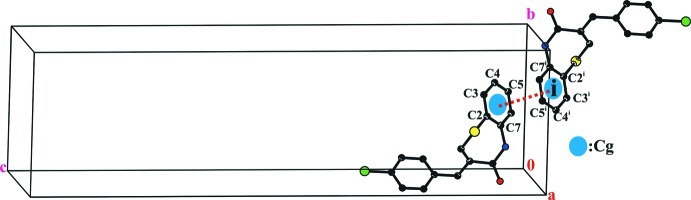
A view of a π—π interaction (dotted lines) in the crystal structure of the title compound. Cg denotes centroid of the C2–C7 benzene ring [Symmetry code: (i) -x, 1 - y, -z].

## supplementary crystallographic information

### Comment

The title compound is used as an intermediate for the synthesis of dosulepin, which is an anti-depressant of the tricyclic family. Dosulepin prevents reabsorbing of serotonin and noradrenaline in the brain, helps to prolong the mood lightening effect of any released noradrenaline and serotonin, thus relieving depression. The dibenzo[c,e]thiazepin derivatives exhibit chiroptical properties (Tomascovic *et al.*, 2000). Dibenzo[b,e]thiazepin-5,5-dioxide derivatives possess anti-histaminic and anti-allergenic activities (Rajsner *et al.*, 1971). Benzene thiazepin derivatives are identified as a new type of effective anti-histaminic compounds (Metys *et al.*, 1965). In view of this biological importance, the crystal structure of the title compound has been carried out and the results are presented here.

Fig. 1. shows the seven membered thiazepine ring (N1/S1/C1/C2/C7/C8/C9) to adopt a twisted-boat conformation as indicated by puckering parameters (Cremer & Pople, 1975) QT = 1.0015 (16) Å, θ_2_ = 74.1 (1)°, φ_2_ = 177.3 (1)° and φ_3_ = 177.6 (4)°. The dihedral angle between the benzothiazepin ring system and the benzene ring is 50.2 (1)°. The geometric parameters of the title molecule agree well with those reported for similar structures (Sridevi *et al.*, 2011; Sabari *et al.*, 2011).

In the crystal packing (Fig. 2), centrosymmetrically related molecules are linked by N1—H1···O1 hydrogen bonds into cyclic *R_2_^2^*(8) dimers (Bernstein *et al.*, 1995). The crystal packing (Fig. 3) is further stabilized by intermolecular π—π interactions with a *Cg*—*Cg*^i^ separation of 3.763 (1) Å [*Cg* is the centroid of the C2–C7 benzene ring, symmetry code as in Fig. 3].

### Experimental

A mixture of (*Z*)-methyl 2-(bromomethyl)-3-(4-chlorophenyl)acrylate (2 mmol) and *o*-aminothiophenol (2 mmol) in the presence of potassium *tert*-butoxide (4.8 mmol) in dry THF (10 ml) was stirred at room temperature for 1 h. After the completion of the reaction as indicated by TLC, the reaction mixture was concentrated and the resulting crude mass was diluted with water (20 ml) and extracted with ethyl acetate (3 × 20 ml). The organic layer was washed with brine (2 × 20 ml) and dried over anhydrous sodium sulfate. The organic layer was concentrated, which successfully provided the crude final product. The final product was purified by column chromatography on silica gel to afford the title compound in good yield (43%).

### Refinement

H atoms were positioned geometrically, with C—H = 0.93–0.97 Å and N—H = 0.86 Å, and constrained to ride on their parent atoms, with *U*_iso_(H) = 1.2*U*_eq_(N,C).

### Crystal data

**Table:**

C_16_H_12_ClNOS	*F*(000) = 1248
*M**_r_* = 301.78	*D*_x_ = 1.406 Mg m^−^^3^
Orthorhombic, *P**b**c**a*	Mo *K*α radiation, λ = 0.71073 Å
Hall symbol: -P 2ac 2ab	Cell parameters from 3130 reflections
*a* = 9.0486 (3) Å	θ = 2.4–27.0°
*b* = 9.4105 (3) Å	µ = 0.41 mm^−^^1^
*c* = 33.4876 (10) Å	*T* = 293 K
*V* = 2851.53 (16) Å^3^	Block, colourless
*Z* = 8	0.23 × 0.21 × 0.15 mm

### Data collection

**Table:**

Bruker APEXII CCD diffractometer	3099 independent reflections
Radiation source: fine-focus sealed tube	2382 reflections with *I* > 2σ(*I*)
Graphite monochromator	*R*_int_ = 0.029
Detector resolution: 10.0 pixels mm^-1^	θ_max_ = 27.0°, θ_min_ = 2.4°
ω scans	*h* = −11→11
Absorption correction: multi-scan (*SADABS*; Sheldrick, 1996)	*k* = −10→11
*T*_min_ = 0.910, *T*_max_ = 0.941	*l* = −42→29
14850 measured reflections	

### Refinement

**Table:**

Refinement on *F*^2^	Secondary atom site location: difference Fourier map
Least-squares matrix: full	Hydrogen site location: inferred from neighbouring sites
*R*[*F*^2^ > 2σ(*F*^2^)] = 0.047	H-atom parameters constrained
*wR*(*F*^2^) = 0.118	*w* = 1/[σ^2^(*F*_o_^2^) + (0.0432*P*)^2^ + 1.9074*P*] where *P* = (*F*_o_^2^ + 2*F*_c_^2^)/3
*S* = 1.03	(Δ/σ)_max_ < 0.001
3099 reflections	Δρ_max_ = 0.35 e Å^−^^3^
182 parameters	Δρ_min_ = −0.45 e Å^−^^3^
0 restraints	Extinction correction: *SHELXL97* (Sheldrick, 2008), Fc^*^=kFc[1+0.001xFc^2^λ^3^/sin(2θ)]^-1/4^
Primary atom site location: structure-invariant direct methods	Extinction coefficient: 0.0057 (6)

### Special details

**Table:**

Geometry. All e.s.d.'s (except the e.s.d. in the dihedral angle between two l.s. planes) are estimated using the full covariance matrix. The cell e.s.d.'s are taken into account individually in the estimation of e.s.d.'s in distances, angles and torsion angles; correlations between e.s.d.'s in cell parameters are only used when they are defined by crystal symmetry. An approximate (isotropic) treatment of cell e.s.d.'s is used for estimating e.s.d.'s involving l.s. planes.
Refinement. Refinement of *F*^2^ against ALL reflections. The weighted *R*-factor *wR* and goodness of fit *S* are based on *F*^2^, conventional *R*-factors *R* are based on *F*, with *F* set to zero for negative *F*^2^. The threshold expression of *F*^2^ > σ(*F*^2^) is used only for calculating *R*-factors(gt) *etc*. and is not relevant to the choice of reflections for refinement. *R*-factors based on *F*^2^ are statistically about twice as large as those based on *F*, and *R*- factors based on ALL data will be even larger.

### Fractional atomic coordinates and isotropic or equivalent isotropic displacement parameters (Å^2^)

**Table:**

	*x*	*y*	*z*	*U*_iso_*/*U*_eq_	
C1	0.0719 (3)	0.1514 (2)	0.12513 (6)	0.0476 (5)	
H1A	0.0655	0.1466	0.1528	0.057*	
C2	0.0392 (2)	0.3707 (2)	0.07497 (6)	0.0435 (5)	
C3	0.0021 (3)	0.5128 (3)	0.08041 (7)	0.0580 (7)	
H3	0.0457	0.5643	0.1010	0.070*	
C4	−0.0985 (3)	0.5777 (3)	0.05567 (8)	0.0618 (7)	
H4	−0.1210	0.6733	0.0592	0.074*	
C5	−0.1654 (3)	0.5023 (2)	0.02586 (8)	0.0518 (6)	
H5	−0.2335	0.5466	0.0092	0.062*	
C6	−0.1323 (2)	0.3610 (2)	0.02049 (7)	0.0434 (5)	
H6	−0.1789	0.3098	0.0004	0.052*	
C7	−0.0300 (2)	0.2947 (2)	0.04485 (6)	0.0373 (4)	
C8	0.0158 (2)	0.0388 (2)	0.06027 (6)	0.0399 (5)	
C9	0.0015 (2)	0.0533 (2)	0.10436 (6)	0.0416 (5)	
C10	−0.0895 (3)	−0.0641 (3)	0.12279 (7)	0.0555 (6)	
H10A	−0.1879	−0.0612	0.1114	0.067*	
H10B	−0.0457	−0.1547	0.1156	0.067*	
C11	−0.1027 (3)	−0.0572 (2)	0.16758 (7)	0.0489 (6)	
C12	−0.0147 (3)	−0.1404 (3)	0.19132 (8)	0.0590 (6)	
H12	0.0524	−0.2022	0.1794	0.071*	
C13	−0.0233 (3)	−0.1346 (3)	0.23246 (8)	0.0668 (7)	
H13	0.0367	−0.1922	0.2482	0.080*	
C14	−0.1208 (3)	−0.0436 (3)	0.24963 (8)	0.0639 (7)	
C15	−0.2101 (3)	0.0408 (3)	0.22704 (9)	0.0733 (8)	
H15	−0.2766	0.1028	0.2391	0.088*	
C16	−0.2004 (3)	0.0329 (3)	0.18587 (8)	0.0664 (7)	
H16	−0.2614	0.0899	0.1703	0.080*	
N1	0.0078 (2)	0.15261 (18)	0.03612 (5)	0.0405 (4)	
H1	0.0290	0.1363	0.0115	0.049*	
O1	0.0322 (2)	−0.08062 (16)	0.04596 (4)	0.0551 (4)	
S1	0.17684 (7)	0.29044 (7)	0.104528 (18)	0.0568 (2)	
Cl1	−0.13112 (12)	−0.03656 (12)	0.30146 (2)	0.1046 (4)	

### Atomic displacement parameters (Å^2^)

**Table:**

	*U*^11^	*U*^22^	*U*^33^	*U*^12^	*U*^13^	*U*^23^
C1	0.0518 (13)	0.0530 (13)	0.0379 (11)	−0.0069 (11)	−0.0027 (10)	0.0015 (10)
C2	0.0495 (13)	0.0381 (12)	0.0428 (11)	−0.0122 (10)	0.0066 (9)	−0.0021 (9)
C3	0.0789 (18)	0.0400 (13)	0.0552 (14)	−0.0191 (13)	0.0111 (13)	−0.0109 (11)
C4	0.0796 (19)	0.0336 (12)	0.0721 (17)	0.0004 (12)	0.0225 (15)	−0.0012 (12)
C5	0.0508 (14)	0.0379 (12)	0.0667 (15)	0.0020 (11)	0.0135 (12)	0.0109 (11)
C6	0.0418 (11)	0.0363 (12)	0.0522 (12)	−0.0045 (9)	0.0039 (10)	0.0036 (9)
C7	0.0389 (11)	0.0328 (10)	0.0400 (10)	−0.0049 (9)	0.0067 (8)	−0.0009 (8)
C8	0.0440 (12)	0.0361 (12)	0.0396 (10)	0.0032 (9)	−0.0035 (9)	−0.0018 (9)
C9	0.0469 (12)	0.0398 (12)	0.0380 (10)	0.0010 (10)	−0.0028 (9)	0.0018 (9)
C10	0.0696 (16)	0.0478 (13)	0.0492 (13)	−0.0110 (12)	−0.0046 (12)	0.0040 (11)
C11	0.0515 (14)	0.0455 (13)	0.0497 (12)	−0.0084 (11)	−0.0005 (10)	0.0095 (10)
C12	0.0615 (16)	0.0559 (15)	0.0595 (14)	0.0044 (13)	−0.0010 (12)	0.0090 (12)
C13	0.0687 (18)	0.0723 (18)	0.0593 (15)	−0.0046 (15)	−0.0117 (14)	0.0210 (14)
C14	0.0653 (17)	0.0791 (18)	0.0472 (13)	−0.0228 (15)	0.0080 (12)	0.0081 (13)
C15	0.0719 (19)	0.078 (2)	0.0698 (17)	0.0061 (16)	0.0219 (15)	0.0040 (15)
C16	0.0631 (17)	0.0714 (18)	0.0645 (16)	0.0115 (14)	0.0025 (13)	0.0179 (14)
N1	0.0527 (11)	0.0347 (9)	0.0342 (8)	0.0024 (8)	0.0011 (8)	−0.0025 (7)
O1	0.0836 (12)	0.0365 (8)	0.0453 (8)	0.0120 (8)	−0.0058 (8)	−0.0030 (7)
S1	0.0541 (4)	0.0654 (4)	0.0509 (3)	−0.0224 (3)	−0.0093 (3)	0.0030 (3)
Cl1	0.1122 (7)	0.1522 (9)	0.0494 (4)	−0.0371 (7)	0.0121 (4)	0.0051 (5)

### Geometric parameters (Å, º)

**Table:**

C1—C9	1.320 (3)	C8—C9	1.488 (3)
C1—S1	1.758 (2)	C9—C10	1.509 (3)
C1—H1A	0.9300	C10—C11	1.506 (3)
C2—C7	1.386 (3)	C10—H10A	0.9700
C2—C3	1.391 (3)	C10—H10B	0.9700
C2—S1	1.761 (2)	C11—C16	1.370 (4)
C3—C4	1.374 (4)	C11—C12	1.371 (3)
C3—H3	0.9300	C12—C13	1.381 (4)
C4—C5	1.366 (4)	C12—H12	0.9300
C4—H4	0.9300	C13—C14	1.357 (4)
C5—C6	1.374 (3)	C13—H13	0.9300
C5—H5	0.9300	C14—C15	1.363 (4)
C6—C7	1.382 (3)	C14—Cl1	1.739 (3)
C6—H6	0.9300	C15—C16	1.383 (4)
C7—N1	1.411 (3)	C15—H15	0.9300
C8—O1	1.231 (2)	C16—H16	0.9300
C8—N1	1.344 (3)	N1—H1	0.8600
			
C9—C1—S1	125.10 (17)	C11—C10—H10A	108.6
C9—C1—H1A	117.5	C9—C10—H10A	108.6
S1—C1—H1A	117.5	C11—C10—H10B	108.6
C7—C2—C3	118.8 (2)	C9—C10—H10B	108.6
C7—C2—S1	120.49 (17)	H10A—C10—H10B	107.6
C3—C2—S1	120.64 (18)	C16—C11—C12	118.0 (2)
C4—C3—C2	120.6 (2)	C16—C11—C10	121.6 (2)
C4—C3—H3	119.7	C12—C11—C10	120.5 (2)
C2—C3—H3	119.7	C11—C12—C13	121.5 (3)
C5—C4—C3	120.2 (2)	C11—C12—H12	119.2
C5—C4—H4	119.9	C13—C12—H12	119.2
C3—C4—H4	119.9	C14—C13—C12	119.0 (3)
C4—C5—C6	120.1 (2)	C14—C13—H13	120.5
C4—C5—H5	120.0	C12—C13—H13	120.5
C6—C5—H5	120.0	C13—C14—C15	121.2 (3)
C5—C6—C7	120.4 (2)	C13—C14—Cl1	118.8 (2)
C5—C6—H6	119.8	C15—C14—Cl1	120.0 (2)
C7—C6—H6	119.8	C14—C15—C16	118.9 (3)
C6—C7—C2	119.92 (19)	C14—C15—H15	120.5
C6—C7—N1	117.91 (18)	C16—C15—H15	120.5
C2—C7—N1	122.02 (19)	C11—C16—C15	121.3 (3)
O1—C8—N1	119.98 (18)	C11—C16—H16	119.3
O1—C8—C9	118.70 (18)	C15—C16—H16	119.3
N1—C8—C9	121.31 (18)	C8—N1—C7	130.05 (17)
C1—C9—C8	123.0 (2)	C8—N1—H1	115.0
C1—C9—C10	124.05 (19)	C7—N1—H1	115.0
C8—C9—C10	112.71 (18)	C1—S1—C2	99.08 (10)
C11—C10—C9	114.76 (19)		
			
C7—C2—C3—C4	−1.7 (3)	C9—C10—C11—C16	79.7 (3)
S1—C2—C3—C4	175.82 (19)	C9—C10—C11—C12	−99.3 (3)
C2—C3—C4—C5	1.3 (4)	C16—C11—C12—C13	0.1 (4)
C3—C4—C5—C6	0.0 (4)	C10—C11—C12—C13	179.1 (2)
C4—C5—C6—C7	−0.7 (3)	C11—C12—C13—C14	−0.4 (4)
C5—C6—C7—C2	0.2 (3)	C12—C13—C14—C15	0.4 (4)
C5—C6—C7—N1	−175.39 (19)	C12—C13—C14—Cl1	−179.9 (2)
C3—C2—C7—C6	1.0 (3)	C13—C14—C15—C16	0.0 (4)
S1—C2—C7—C6	−176.59 (15)	Cl1—C14—C15—C16	−179.7 (2)
C3—C2—C7—N1	176.41 (19)	C12—C11—C16—C15	0.3 (4)
S1—C2—C7—N1	−1.2 (3)	C10—C11—C16—C15	−178.7 (2)
S1—C1—C9—C8	6.9 (3)	C14—C15—C16—C11	−0.3 (4)
S1—C1—C9—C10	−179.25 (19)	O1—C8—N1—C7	170.5 (2)
O1—C8—C9—C1	135.4 (2)	C9—C8—N1—C7	−8.1 (3)
N1—C8—C9—C1	−46.0 (3)	C6—C7—N1—C8	−131.7 (2)
O1—C8—C9—C10	−39.0 (3)	C2—C7—N1—C8	52.8 (3)
N1—C8—C9—C10	139.5 (2)	C9—C1—S1—C2	58.0 (2)
C1—C9—C10—C11	3.5 (3)	C7—C2—S1—C1	−60.21 (19)
C8—C9—C10—C11	177.9 (2)	C3—C2—S1—C1	122.26 (19)

### Hydrogen-bond geometry (Å, º)

**Table:**

*D*—H···*A*	*D*—H	H···*A*	*D*···*A*	*D*—H···*A*
N1—H1···O1^i^	0.86	2.07	2.854 (2)	151

Symmetry code: (i) −*x*, −*y*, −*z*.
